# Phylogenetic Relationships in Orobanchaceae Inferred From Low-Copy Nuclear Genes: Consolidation of Major Clades and Identification of a Novel Position of the Non-photosynthetic *Orobanche* Clade Sister to All Other Parasitic Orobanchaceae

**DOI:** 10.3389/fpls.2019.00902

**Published:** 2019-07-16

**Authors:** Xi Li, Tao Feng, Chris Randle, Gerald M. Schneeweiss

**Affiliations:** ^1^Department of Botany and Biodiversity Research, University of Vienna, Vienna, Austria; ^2^Wuhan Botanical Garden, Chinese Academy of Sciences, Wuhan, China; ^3^Department of Biological Sciences, Sam Houston State University, Huntsville, TX, United States

**Keywords:** low-copy nuclear genes, Orobanchaceae, parasitic plants, phylogeny, PPR genes

## Abstract

Molecular phylogenetic analyses have greatly advanced our understanding of phylogenetic relationships in Orobanchaceae, a model system to study parasitism in angiosperms. As members of this group may lack some genes widely used for phylogenetic analysis and exhibit varying degrees of accelerated base substitution in other genes, relationships among major clades identified previously remain contentious. To improve inferences of phylogenetic relationships in Orobanchaceae, we used two pentatricopeptide repeat (PPR) and three low-copy nuclear (LCN) genes, two of which have been developed for this study. Resolving power and level of support strongly differed among markers. Despite considerable incongruence among newly and previously sequenced markers, monophyly of major clades identified in previous studies was confirmed and, especially in analyses of concatenated data, strongly supported after the exclusion of a small group of East Asian genera (*Pterygiella* and *Phtheirospermum*) from the *Euphrasia-Rhinanthus* clade. The position of the *Orobanche* clade sister to all other parasitic Orobanchaceae may indicate that the shift to holoparasitism occurred early in the evolution of the family. Although well supported in analyses of concatenated data comprising ten loci (five newly and five previously sequenced), relationships among major clades, most prominently the *Striga-Alectra* clade, the *Euphrasia-Rhinanthus* clade, and the *Castilleja-Pedicularis* clade, were uncertain because of strongly supported incongruence also among well-resolving loci. Despite the limitations of using a few selected loci, congruence among markers with respect to circumscription of major clades of Orobanchaceae renders those frameworks for detailed, species-level, phylogenetic studies.

## Introduction

Parasitic plants attach to other plants via a specialized organ, the haustorium, to obtain nutrients and water from their hosts (Kuijt, 1969). This renders parasitic plants of interest not only for plant scientists, who investigate structural, physiological, and molecular adaptations of parasitism (dePamphilis and Palmer, 1990; Cubero and Moreno, 1996; Joel et al., 2013) but also for farmers and applied scientists, because some parasitic plants are serious agricultural pests that can cause major yield losses (Parker and Riches, 1993). Within angiosperms, parasitism has evolved at least twelve times independently (Schneeweiss, 2013) and around 1% of all angiosperm species are parasitic plants, i.e., c. 4,500 species in about 20–30 families (Nickrent et al., 1998; Nickrent, 2019).

An excellent model system for studying the evolution of parasitism in plants is the family Orobanchaceae. Orobanchaceae is the largest parasitic family, comprising more than 2,000 species in about 90–115 genera (McNeal et al., 2013; Schneeweiss, 2013), and includes the full range of nutritional dependency from non-parasitic via photosynthetic parasitic (hemiparasitic) to non-photosynthetic parasitic (holoparasitic). Whereas parasitism has evolved only once in Orobanchaceae, the transition from hemi- to holoparasitism has occurred multiple times (Schneeweiss, 2013).

Molecular phylogenetic analyses have greatly advanced our understanding of phylogenetic relationships of Orobanchaceae. These have led to a greatly expanded circumscription of the family from the traditional Orobanchaceae, which previously comprised the exclusively holoparasitic *Orobanche* and a few related genera only (Beck-Mannagetta, 1930), to include all hemiparasites and the few holoparasites formerly placed in Scrophulariaceae (Young et al., 1999; Wolfe et al., 2005; Bennett and Mathews, 2006; McNeal et al., 2013). The sister group to parasitic Orobanchaceae is the Asian non-parasitic genus *Lindenbergia*, now commonly included in the thus-broadened Orobanchaceae (Young et al., 1999; Wolfe et al., 2005; Bennett and Mathews, 2006; Park et al., 2008; McNeal et al., 2013; but see Fischer, 2004). Only recently Rehmanniaceae (including two non-parasitic genera, *Rehmannia* and *Triaenophora*), the sister to Orobanchaceae (Albach et al., 2009; Xia et al., 2009), has been merged with Orobanchaceae as well (Angiosperm Phylogeny Group, 2016). The second major impact of molecular phylogenetic data concerns the identification of several major lineages within Orobanchaceae (dePamphilis et al., 1997; Wolfe and dePamphilis, 1998; Young et al., 1999; Bennett and Mathews, 2006; McNeal et al., 2013). These are the non-parasitic *Lindenbergia* clade; the small, hemiparasitic *Cymbaria-Siphonostegia* clade; the exclusively holoparasitic *Orobanche* clade; the exclusively hemiparasitic *Castilleja-Pedicularis* clade; the nearly exclusively hemiparasitic *Euphrasia-Rhinanthus* clade; the mainly tropical, mostly hemiparasitic *Striga-Alectra* clade; and the single genus *Brandisia* (Schneeweiss, 2013).

Despite these advances, our understanding of phylogenetic relationships within Orobanchaceae is hampered by two major shortcomings. The first is that about one third of the genera, especially those with tropical distributions, have not been studied yet using molecular phylogenetic tools. The second, which is the focus of this study, is that relationships among major clades were either poorly resolved (e.g., the position of *Brandisia*: Bennett and Mathews, 2006; McNeal et al., 2013) or suffered from, partly well supported, incongruent results from different markers (e.g., the position of *Lindenbergia* differed between two phytochrome genes: McNeal et al., 2013) or even from different data sets of the same marker (e.g., relationships among the *Castilleja-Pedicularis* clade, the *Euphrasia-Rhinanthus* clade, and the *Striga-Alectra* clade inferred from phytochrome A data were swapped in the study of McNeal et al., 2013, compared to that of Bennett and Mathews, 2006). These issues may be due to insufficient phylogenetic signal and/or marker-specific problems, such as substitution rate variation of plastid genes evolving under relaxed functional constraints (dePamphilis et al., 1997; Wolfe and dePamphilis, 1998; Wicke et al., 2013, 2016), paralogy issues in multi-copy genes such as ITS (Álvarez and Wendel, 2003) or in low-copy genes (Zimmer and Wen, 2012) such as phytochrome genes (Bennett and Mathews, 2006; McNeal et al., 2013). Evidently, additional nuclear low-copy markers, although no panacea for resolving all relationships, are needed to obtain a robust phylogenetic framework of Orobanchaceae.

A number of nuclear genes have recently been used to improve molecular phylogenetic analyses in plants. These include low-copy nuclear (LCN) Conserved Ortholog Set (COS) genes (Sang, 2002; Li M. et al., 2008; Duarte et al., 2010; Zhang et al., 2012; Zimmer and Wen, 2012, 2015; Babineau et al., 2013; Latvis et al., 2017) as well as multi-gene families, most notably pentatricopeptide repeat (PPR) genes (Yuan et al., 2009, 2010; Crowl et al., 2014). Members of the PPR protein family are sequence-specific RNA-binding proteins functioning in gene expression of chloroplasts and mitochondria (O’Toole et al., 2008; Barkan and Small, 2014), with over 400 members in the genomes of most plants sampled thus far (Yuan et al., 2010). Screening the model plants rice (*Oryza sativa*) and *Arabidopsis thaliana*, Yuan et al. (2010) found 127 PPR genes to be single copy, of which five were used to resolve phylogenetic relationships in selected Verbenaceae (Yuan et al., 2010). The applicability of LCN genes may decrease at deeper phylogenetic depth (e.g., of 274 LCN loci screened in Fabaceae by Choi et al., 2006, only ten markers were suitable at the family level), which may explain why beyond phytochrome genes (PHYA and PHYB) no LCN locus has been applied across the entire Orobanchaceae (but see Latvis et al., 2017, for a list of primers from a number of single-copy nuclear loci).

In this study, we analyze two PPR genes successfully applied in other angiosperms (Yuan et al., 2010) as well as three LCN loci, two newly established here, to infer phylogenetic relationships of major lineages within Orobanchaceae. We analyze these PPR and LCN loci both individually and jointly with previously used markers (plastid DNA, nuclear ITS, PHYA and PHYB). Specifically, we want to solve remaining uncertainties concerning (i) the unclear positions of *Brandisia* and the *Cymbaria-Siphonostegia* clade, (ii) the ambiguous support for monophyly of the *Orobanche* clade, and (iii) the contradicting relationships among the *Castilleja-Pedicularis* clade, the *Euphrasia-Rhinanthus* clade, and the *Striga-Alectra* clade inferred previously (Bennett and Mathews, 2006; McNeal et al., 2013). Additionally, we also want to assess the suitability of these markers at lower taxonomic levels using *Odontites* (from the *Euphrasia-Rhinanthus* clade), where recent phylogenetic work has revealed strong discrepancies among markers (Pinto-Carrasco et al., 2017; Gaudeul et al., 2018).

## Materials and Methods

### Plant Material

We included 56 species of 31 genera of Orobanchaceae (Table 1 and Supplementary Table S1). These taxa covered all major clades identified in previous studies (Bennett and Mathews, 2006; McNeal et al., 2013). Compared to McNeal et al. (2013), the most comprehensive phylogenetic study of Orobanchaceae to date, we have overall sparser taxon sampling, especially in the tropical *Striga-Alectra* clade and the *Euphrasia-Pedicularis* clade, but we include the following previously unsampled genera: *Macrosyringion*, *Nothobartsia*, *Odontitella*, *Phtheirospermum* (except *Phtheirospermum japonicum*), *Rehmannia*, and *Triaenophora*.

**TABLE 1:** List of taxa and source of sequence information (for details see Supplementary Table S1).

**Taxon**	**AT1G04780**	**AT1G14610**	***Agt1***	**AT2G37230**	**AT1G09680**	***PHYA***	***PHYB***	**ITS**	***matK***	***rps2***
***Rehmannia-Triaenophora* Clade**										
*Rehmannia piasezkii*	+	+	+	+	+	+	+	+^GB^	+	+^GB^
*Triaenophora shennongjiaensis*	+	+	+	+	+	+		+^GB^	+	+^GB^
***Lindenbergia* Clade**										
*Lindenbergia muraria*	+	+			+	+^GB^	+^GB^	+^GB^	+^GB^	+^GB^
*Lindenbergia philippensis*	+	+	+	+	+	+^GB^	+^GB^	+^GB^	+^GB^	+^GB^
***Cymbaria-Siphonostegia* Clade**										
*Bungea trifida*	+	+	+	+	+	+^GB^	+	+^GB^	+^GB^	+^GB^
*Schwalbea americana*	+	+	+	+	+	+^GB^	+^GB^	+^GB^	+^GB^	+^GB^
***Orobanche* Clade**										
*Boschniakia himalaica*	+			+	+	+^GB^	+^GB^	+^GB^	+^GB^	+^GB^
*Cistanche phelypaea*	+	+		+	+			+^GB^	+^GB^	+^GB^
*Cistanche tubulosa*	+	+		+	+			+^GB^	+^GB^	+^GB^
*Epifagus virginiana*	+	+	+	+		+^GB^	+^GB^	+^GB^	+^GB^	+^GB^
*Orobanche caryophyllacea*	+	+		+	+		+	+^GB^	+^GB^	+^GB^
*Orobanche flava*	+	+	+	+	+	+	+	+^GB^	+	+^GB^
*Orobanche gracilis*	+	+		+	+	+^GB^	+	+^GB^	+^GB^	+^GB^
*Orobanche lycoctoni*	+	+		+	+	+	+	+^GB^	+	
*Phelipanche aegyptiaca*	+^PPGP^	+^PPGP^	+^PPGP^	+^PPGP^	+^PPGP^			+^*GB*^	+^*GB*^	+^*GB*^
*Phelipanche arenaria*	+	+						+^*GB*^		+^*GB*^
**Incertae sedis**										
*Brandisia hancei*	+	+	+	+	+	+^*GB*^	+^*GB*^	+^*GB*^	+^*GB*^	+^*GB*^
***Pterygiella* Clade**										
*Phtheirospermum tenuisectum*	+	+		+			+	+^*GB*^	+^*GB*^	+
*Pterygiella cylindrica*	+	+	+	+	+	+	+	+^*GB*^	+^*GB*^	+
*Pterygiella duclouxii*	+	+	+	+	+	+	+	+^*GB*^	+^*GB*^	+
***Castilleja-Pedicularis* Clade**										
*Pedicularis aspleniifolia*	+	+	+	+	+			+^*GB*^		
*Pedicularis decora*	+		+	+	+			+^*GB*^	+^*GB*^	
*Pedicularis densispica*	+	+	+	+	+	+^*GB*^	+^*GB*^	+^*GB*^	+^*GB*^	+^*GB*^
*Pedicularis elwesii*	+	+	+	+	+	+	+^*GB*^	+^*GB*^	+^*GB*^	+^*GB*^
*Pedicularis lachnoglossa*	+	+	+	+	+			+^*GB*^	+^*GB*^	
*Pedicularis rex*		+			+			+^*GB*^	+^*GB*^	
*Pedicularis rostrato spicata*	+	+	+	+	+					
*Pedicularis verticillata*	+		+	+	+	+		+^*GB*^	+^*GB*^	
*Triphysaria pusilla*	+^PPGP^		+^PPGP^		+^PPGP^	+^*GB*^	+^*GB*^	+^*GB*^		
*Triphysaria versicolor*	+^PPGP^	+^PPGP^	+^PPGP^	+^PPGP^	+^PPGP^			+^*GB*^	+^*GB*^	+^*GB*^
***Euphrasia-Rhinanthus* Clade**										
*Bellardia trixago*		+		+	+	+^*GB*^	+^*GB*^	+^*GB*^	+^*GB*^	+^*GB*^
*Euphrasia frigida*	+	+	+	+				+^*GB*^	+^*GB*^	
*Euphrasia sinuata*		+	+	+	+					
*Euphrasia stricta*		+		+		+^*GB*^	+	+^*GB*^	+^*GB*^	+^*GB*^
*Lathraea squamaria*	+	+	+^*GB*^	+	+	+^*GB*^	+^*GB*^		+^*GB*^	+^*GB*^
*Macrosyringion longiflorum*	+	+	+	+				+^*GB*^	+^*GB*^	
*Melampyrum sylvaticum*		+	+^*GB*^	+	+	+^*GB*^		+^*GB*^	+^*GB*^	+^*GB*^
*Nothobartsia asperrima*	+	+	+	+	+			+^*GB*^	+^*GB*^	
*Odontitella virgata*	+	+	+	+	+			+^*GB*^	+^*GB*^	
*Odontites bolligeri*	+	+	+	+	+			+^*GB*^	+^*GB*^	
*Odontites cebennensis*	+	+		+	+			+^*GB*^	+^*GB*^	
*Odontites luteus*	+		+	+	+			+^*GB*^	+^*GB*^	
*Odontites vernus*	+		+	+	+			+^*GB*^	+^*GB*^	
*Odontites viscosus*	+	+		+	+			+^*GB*^	+^*GB*^	
*Parentucellia latifolia*	+	+	+	+		+^*GB*^	+^*GB*^	+^*GB*^	+^*GB*^	+^*GB*^
*Parentucellia viscosa*				+		+^*GB*^	+^*GB*^	+^*GB*^	+^*GB*^	+^*GB*^
*Rhinanthus alectorolophus*	+	+	+^*GB*^	+	+	+^*GB*^	+^*GB*^	+^*GB*^	+^*GB*^	+^*GB*^
***Striga-Alectra* Clade**										
*Aeginetia indica*	*+*			+^4^		+^*GB*^	+^*GB*^	+^*GB*^		
*Buchnera americana*	+	+	+	+		+^*GB*^	+^*GB*^	+^*GB*^	+^*GB*^	
*Buchnera hispida*	+	+						+^*GB*^	+^*GB*^	+
*Radamaea montana*	+		+	+^4^	+	+^*GB*^	+^*GB*^	+^*GB*^	+^*GB*^	+^*GB*^
*Striga bilabiata*	+	+^1^	+	+^3^		+^*GB*^	+^*GB*^	+^*GB*^	+^*GB*^	+^*GB*^
*Striga gesnerioides*	+	+^3^	+	+		+^*GB*^	+^*GB*^	+^*GB*^	+^*GB*^	+^*GB*^
*Striga hermonthica*	+^PPGP^	+^PPGP^	+^PPGP^	+^PPGP^	+^PPGP^			+^*GB*^	+^*GB*^	+^*GB*^
**Out-groups**										
*Paulownia* sp.^1^	+^1KP^	+^1KP^	+^1KP^	+^1KP^	+^1KP^	+^*GB*^	+^*GB*^		+^*GB*^	+^*GB*^
*Mimulus guttatus*	+^PZ^	+^PZ^	+^PZ^	+^PZ^	+^PZ^			+^*GB*^	+^*GB*^	+^*GB*^
Total number	50	47	39	51	43	31	30	34	34	31

*+Indicate sequences newly obtained in this study (superscript numbers indicate number of clones, where applicable); +^*GB*^ Indicate sequences from previous studies obtained from GenBank; +^*PPGP*^ indicate sequences (EST libraries or combined builds) obtained from the Parasitic Plant Genome Project (PPGP) database (available from: http://ppgp.huck.psu.edu/; assessed on Feb 27th 2017); +^1KP^ indicate sequences from the 1KP database (http://www.onekp.com/public_data.html; assessed on Feb 27th 2017); +^*PZ*^ sequences from Phytozome 12.1 database (https://phytozome.jgi.doe.gov/pz/portal.html#; assessed on Feb 27th 2017). ^1^Sequences from the 1KP project (+^1KP^) are from *P. fargesii*, the remaining ones (+^*GB*^) are from *P. tomentosa*.*

### Marker Development

Our goal was to establish several low-copy markers that amplify well (ideally without requiring any cloning) across the entire family Orobanchaceae. To this end, we tested both already published and newly developed markers. To retrieve homologous LCN genes from Orobanchaceae, we conducted a BLASTN search (as implemented on the Parasitic Plant Genome Project^1^) on genes from *Arabidopsis* that have been shown to be low-copy in *Arabidopsis*, *Populus*, *Vitis*, and *Oryza* (Duarte et al., 2010) against unigenes from four Orobanchaceae species [*Lindenbergia philippensis*, *Phelipanche* (*Orobanche*) *aegyptiaca*, *Striga hermonthica*, *Triphysaria versicolor*] available from the Parasitic Plant Genome Project^2^ (PPGP, Yang et al., 2015) using an *e*-value of e–10. Of the thus retrieved loci, the 200+ longest ones were retained and aligned separately using Muscle 3.8.31 (Edgar, 2004) available as web-service from EMBL-EBI (McWilliam et al., 2013). We chose two species, for which genomic data are available, as outgroups: *Paulownia fargesii* (Paulowniaceae, the sister-group to Orobanchaceae), whose transcriptome data are available from the 1000 Plants (1KP) project^3^ (see Matasci et al., 2014, for details on this project), and *Erythranthe guttata* (syn. *Mimulus guttatus*, Phrymaceae, sister-group to the clade of Orobanchaceae plus Paulowniaceae), whose genome is available from Phytozome 12.1^4^ (see Hellsten et al., 2013, for details on an earlier version genome annotation). Alignments were edited manually in BioEdit 7.2.1 (Hall, 1999). Primers were designed in conserved regions using Primer Premier 5.0 (Premier Biosoft International, Palo Alto, CA, United States) requiring primer lengths of 15–30 bp, GC contents of 40–60%, melting temperatures of 55–75°C, and avoiding repetitive motifs, hairpins, and the potential for dimer formation.

### DNA Extraction, PCR, and Sequencing

Total genomic DNA was extracted using the DNeasy Plant Mini Kit (Qiagen, Hilden, Germany) following the manufacturer’s instructions. We amplified five PPR genes and 90 LCN genes. Most of those, however, could only be amplified and sequenced with limited success. Specifically, 16 of the 90 LCN genes (14.4%) could be PCR amplified from three to 27 species across the family (Supplementary Table S2), but failed to amplify across the entire family. Five loci gave reliable PCR amplification from at least 30 species of Orobanchaceae. These were the LCN gene *Agt1* using modified forward and reverse primers from Li M. et al. (2008), two LCN genes (AT1G04780 and AT1G14610) identified here and two PPR genes (AT1G09680 and AT2G37230) using primers from Yuan et al. (2010); the primers used (including internal ones, where necessary) are listed in Table 2.

**TABLE 2:** Sequences of primers used in this study.

**Primer**	**Sequence**	**References**
*AT1G09680*		
AT1G09680_180f	ACCRCCCTWTCTCAAGCCATCCAAA	Yuan et al. (2010)
AT1G09680_1760r	TARTCAAGAACAAGCCCTTTCGCAC	Yuan et al. (2010)
AT1G09680_850f	GTTAGTTTCAATACTTTGATGAA	Yuan et al. (2010)
AT1G09680_850r	TTCATCAAAGTATTGAAACTAAC	Yuan et al. (2010)
*AT2G37230*		
AT2G37230_320f	GCCTGGACDACMCGTTTRCAGAA	Yuan et al. (2010)
AT2G37230_1770r	TCRAACAAGCTCTCCATCAC	Yuan et al. (2010)
AT2G37230_1800r	GCYGTCTGAACWCSYCCATCYTC	Yuan et al. (2010)
AT2G37230_512f	GGCAACAARGTYGAGTAAG	This study
AT2G37230_1066r	GATGAGGATTTGTGGGT	This study
*AT1G14610*		
AT1G14610f	RAGGCTAGARGAKGGDAACT	This study
AT1G14610r	AAACTGCCACCAYGARTA	This study
*AT1G04780*		
AT1G04780f	CMCTTCATYTGGCTGTTA	This study
AT1G04780r	TCYGDCGAGTCCATYTTA	This study
AT1G04780r511	GGAGMACCWGCACCATCCAA	This study
*Agt1*		
Agt1f_oro	GATTTCCGCATGGAYGARTGGGG	Modified from Li M. et al. (2008)
Agt1r_oro	CCAYTCCTCCTTCTGASTGCAGTT	Modified from Li M. et al. (2008)

*Primers yielding the longest amplicon are underlined, the remaining primers are internal primers.*

Amplification was done in a volume of 15.8 μL containing 0.3 U of KAPA3G Plant DNA Polymerase (Peqlab, Vienna, Austria), 7 μL of 2× PCR buffer, 0.5 μL of 10 μM primers, 0.7 μL DNA, and 7 μL water. PCR conditions for LCN loci amplification were: denaturation for 4 min at 94°C; 35 cycles each with 30 s at 94°C, 30 s at 48°C, 1 min at 72°C; and final elongation for 10 min at 72°C. For the PPR loci we used the protocol of Yuan et al. (2010). For species not included in previous studies (Bennett and Mathews, 2006; McNeal et al., 2013), we also generated *PHYA*, *PHYB*, *matk*, and *rps2* sequences using primers and PCR conditions described by Li et al. (2016). PCR products were purified using 0.5 μL Exonuclease I and 1 μL FastAP thermo sensitive alkaline phosphatase (Thermo Fisher Scientific, St. Leon-Rot, Germany) following the manufacturer’s protocol. A mixture of 5 μL of purified template, 2 μL trehalose, 1.5 μL sequencing buffer, 0.5 μL of primer (10 μM), and 1 μL BigDye Terminator (Applied Biosystems, Foster City, CA, United States) was used in cycle sequencing. Reactions were purified on Sephadex G-50 Fine (GE Healthcare Bio-Sciences, Uppsala, Sweden) and sequenced on an ABI 3730 DNA Analyzer capillary sequencer (Applied Biosystems). For a few species from the *Striga-Alectra* clade direct sequencing of AT1G14610 and AT2G37230 did not result in clean reads (these samples are indicated in Table 1), and these sequences were cloned. To this end, purified PCR products were run on an agarose gel and target bands were isolated using the Quick Gel Extraction Kit (Invitrogen, Vienna, Austria). All PCR products were ligated to vector pGEM-T (Zoman, Beijing, China) and then were transformed into DH5alpha competent *E. coli*. After blue white screening on LB medium, eight white colonies were checked by colony PCR, and at least three positive colonies were sequenced with primers M13F and M13R.

### Phylogenetic Analyses

Sequences were assembled and edited using SeqMan II 5.05 (DNAStar Inc., Madison, United States). Initial alignments of individual loci were made with Muscle 3.8.31 (Edgar, 2004) using the web-service available from EMBL-EBI (McWilliam et al., 2013) and manually adjusted using BioEdit 7.2.1 (Hall, 1999). Parsimony-informative sites were calculated using PAUP^*^ 4.0a163 (Swofford, 2002). These five loci were analyzed separately as well as concatenated into a matrix containing 56 species. Furthermore, we generated a concatenated alignment of 56 species by combining five loci in this study with five loci used by McNeal et al. (2013), i.e., *PHYA*, *PHYB*, ITS, *matK*, and *rps2.* For all analyses (single markers and concatenated data sets), the best-fit substitution models as well as partitioning schemes for DNA sequence alignments (considering codon positions and introns, where applicable, for each marker) were identified via the Akaike Information Criterion (AIC; Akaike, 1974) using PartitionFinder 1.1.0 (Lanfear et al., 2012), employing the greedy algorithm. We tested those 24 models that are implemented in MrBayes. Maximum likelihood analyses were conducted using RAxML 8.1 (Stamatakis, 2014), employing the fast bootstrap approach (Stamatakis et al., 2008) with 1,000 bootstrap replicates and the GTRGAMMA model. Bayesian inference was done using MrBayes 3.2.3 (Ronquist and Huelsenbeck, 2003) using the partitioning schemes and substitution models identified before (see data matrices available in dryad under doi: 10.5061/dryad.31cf160). Values for all parameters, such as the shape of the gamma distribution or the substitution rates, were estimated during the analysis. Partitions were allowed to evolve under different rates (ratepr = variable). We ran four cold Monte Carlo Markov (MCMC) chains simultaneously starting from different random starting trees for 10 million generations, and sampled trees every 5,000th generation. We used Tracer 1.4 (Rambaut et al., 2018) to check the stability of output parameters from Bayesian analyses (i.e., ESS values of at least 200). After combining 1,800 trees from each run (i.e., after discarding 10% trees as burn-in, when the MCMC chain had reached stationarity, evident from standard deviations of split variances being below 0.01), posterior probabilities were estimated.

Possible discrepancies among phylogenetic relationships inferred from different markers (five newly sequenced here, five taken from McNeal et al., 2013) were visualized using super networks (Huson et al., 2004) as implemented in SplitsTree 4 (Huson and Bryant, 2006). To this end, phylogenetic super networks were obtained from the five newly sequenced loci and from all ten loci, i.e., the five newly sequenced ones plus those used by McNeal et al. (2013), with default parameter settings.

The evolution of parasitism was reconstructed on the maximum likelihood tree from the combined 10 loci using maximum parsimony as implemented in Mesquite 3.51 (Maddison and Maddison, 2018). Under the assumption that holoparasitism (i.e., non-photosynthetic parasitism) can only evolve via hemiparasitism (i.e., photosynthetic parasitism), as suggested by the sequence of genome reduction and gene loss in plastomes of parasitic plants (Wicke et al., 2016), we used ordered parsimony for these reconstructions.

## Results

**FIGURE 1:**
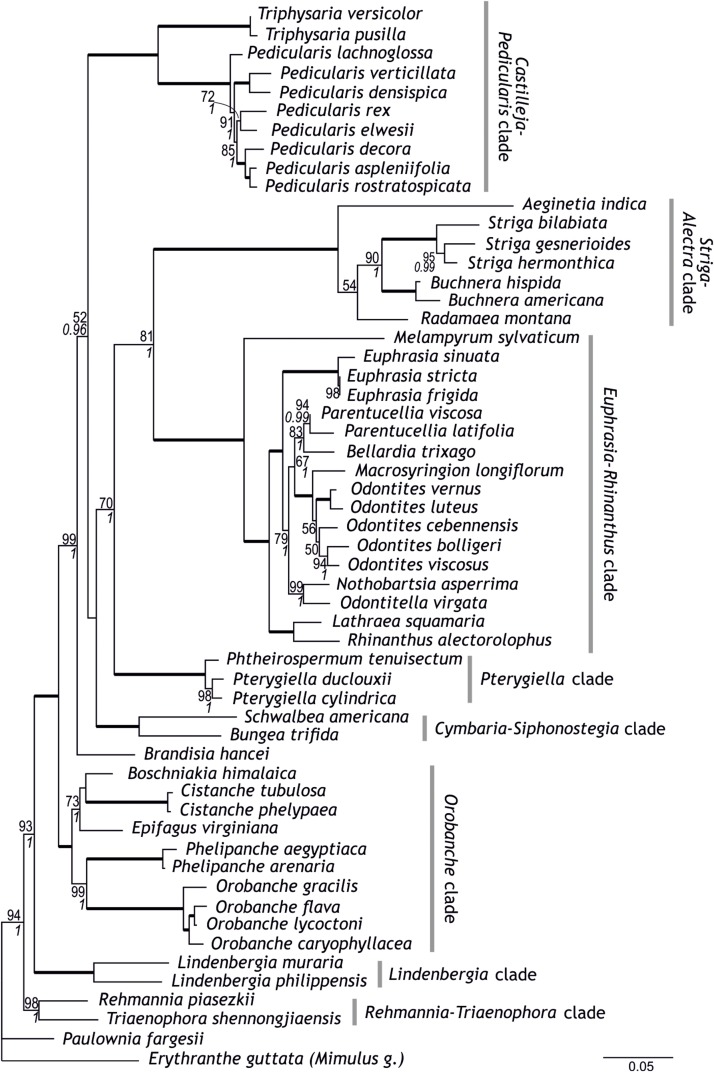
Phylogenetic relationships within Orobanchaceae inferred using maximum likelihood on a combined data set of five loci newly sequenced for this study. Numbers at branches are maximum likelihood bootstrap support values of at least 50 and, in italics, posterior probabilities of at least 0.95; branches with maximum support are indicated by thick lines. Circumscription of major clades within Orobanchaceae is indicated.

**FIGURE 2:**
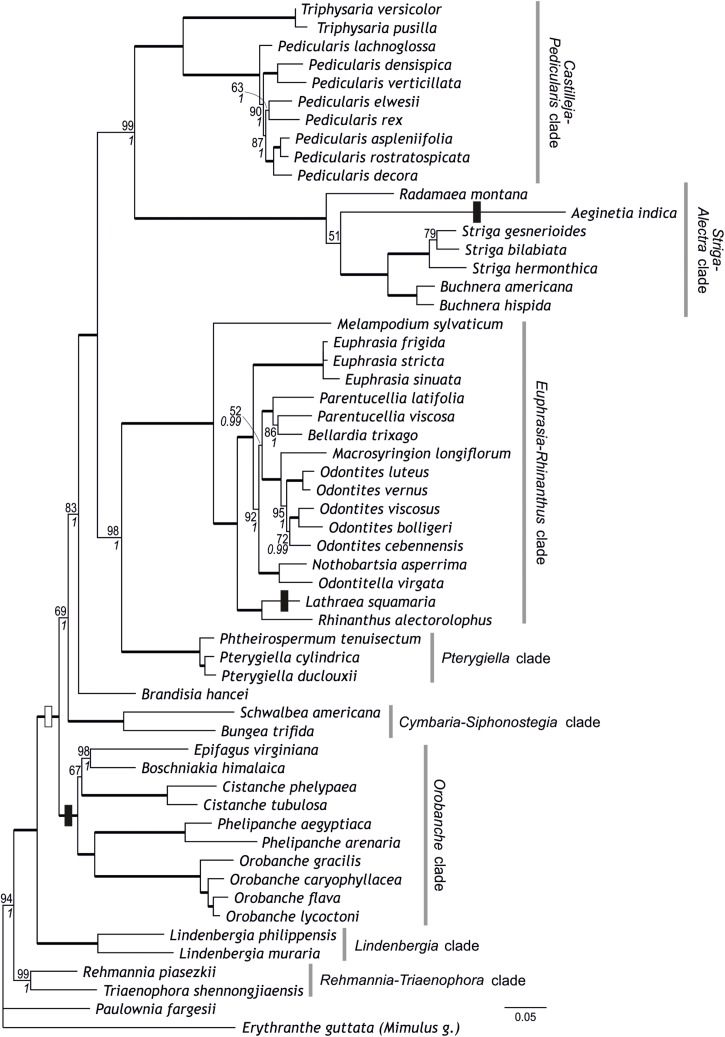
Phylogenetic relationships within Orobanchaceae inferred using maximum likelihood on a combined data set of ten loci. Numbers at branches are maximum likelihood bootstrap support values of at least 50 and, in italics, posterior probabilities of at least 0.95; branches with maximum support are indicated by thick lines. Circumscription of major clades within Orobanchaceae is indicated. The transition to parasitism is indicated by a white box, transitions to holoparasitic (from hemiparasitic ancestors) are indicated by black boxes.

Maximum likelihood and Bayesian analyses resulted in topologically identical trees, with exceptions concerning only weakly supported nodes [bootstrap support (BS) < 0.8 and posterior probabilities (PP) < 0.95]; hence only maximum likelihood trees are shown (Figures 1, 2). All trees (maximum likelihood trees, consensus trees from the Bayesian analyses) are available in the nexus files (available in dryad under doi: 10.5061/dryad.31cf160).

### Single Markers

The five markers were successfully amplified from at least 30 of the 56 taxa (Table 1), thus after adding sequences from other sources (e.g., GenBank) each marker was available for at least 39 of the 56 taxa (Supplementary Table S1). Cloned sequences of a marker from the same species always formed well supported clades (data not shown), and only a single randomly chosen clone per marker and sample was used for final analyses. Alignment lengths of the markers used ranged from 289 bp in *Agt1* to 1508 bp in AT1G09680, the two PPR genes (AT1G09680, AT2G37230) being the longest sequences (Table 3). Introns were present in AT1G14610 and *Agt1*. A few regions, most prominently the intron from *Agt1*, were excluded from phylogenetic analysis because they were not universally alignable across all taxa of the family (Table 3).

**TABLE 3:** Sequences characteristics.

**Locus**	**Sequence length (bp) exon (intron)**	**Alignment length (bp) exon (intron)**	**Number of parsimony-informative sites**
AT1G09680	786–1505 (0)	1508^1^ (0)	734
AT2G37230	391–1380 (0)	1359^2^ (0)	533
AT1G14610	250–362 (55–150)	365 (105^3^)	197
AT1G04780	435–808 (0)	811 (0)	259
*Agt1*	206–289 (220–818)	289 (0^4^)	93

*^1^A sample-specific insertion of 14 bps in *Orobanche flava* has been removed. ^2^An invariant motif of 21 bp at the 3′ terminus sequenced only for three *Pedicularis* species (*P. aspleniifolia*, *P. rostratospicata*, and *P. verticillata*) has been removed. ^3^Sample-specific insertions removed: motifs of 18 and 5 bps, respectively, in *Melampyrum sylvaticum*, a motif of 52 bps in *Striga gesnerioides*. ^4^Intron not universally alignable across all taxa and, therefore, removed.*

The best markers with respect to level of resolution and support were the two PPR genes, AT1G09680, and AT2G37230. AT1G09680, the locus yielding the longest alignment (Table 3), provided good and often well-supported resolution across the entire phylogeny, including the backbone (Supplementary Figure S1). The second PPR gene, AT2G37230, yielding the second longest alignment (Table 3), showed reduced support (especially from maximum likelihood analysis) at the backbone, but usually high support among genera and species, except the *Euphrasia-Rhinanthus* clade (Supplementary Figure S2). Conflicts between the PPR genes concerned, for instance, the placement of the *Cymbaria-Siphonostegia* clade and of *Brandisia*, which received moderate to (especially in Bayesian analysis, if taking posterior probabilities of at least 0.95 into account) high support. The LCN loci AT1G14610 (Supplementary Figure S3) and AT1G04780 (Supplementary Figure S4), which have never been used in any phylogenetic study before, showed poor resolution at the backbone, but better and usually well-supported resolution among genera and species at least in some clades, such as the *Castilleja-Pedicularis* clade, the *Euphrasia-Rhinanthus* clade, or the *Striga-Alectra* clade (Supplementary Figures S3, S4). The locus yielding the shortest alignment (Table 3), *Agt1*, provided poor resolution at all levels in all clades except the *Striga-Alectra* clade (Supplementary Figure S5).

Single markers usually recovered the major clades identified previously (McNeal et al., 2013). Exceptions were the *Orobanche* clade inferred as polyphyletic, though not supported (BS < 50, PP < 0.5), by AT1G14610 data (Supplementary Figure S3), the *Cymbaria-Siphonostegia* clade inferred as polyphyletic, though not supported (BS < 50, PP < 0.5), by AT1G04780 (Supplementary Figure S4), and the *Castilleja-Pedicularis* clade inferred as paraphyletic, though not supported (BS < 50, PP < 0.5), by *Agt1* (Supplementary Figure S5). The clade comprising *Rehmannia* and *Triaenophora*, henceforth referred to as *Rehmannia-Triaenophora* clade, was inferred as non-monophyletic not only by the two short markers AT1G04780 (Supplementary Figure S4) and *Agt1* (Supplementary Figure S5), but also by one of the PPR genes (AT2G37230, Supplementary Figure S2), but in none of these cases did the lack of monophyly receive sufficient support. Congruently, a clade of several *Pterygiella* species and *Phtheirospermum tenuisectum*, the *Pterygiella* clade, was identified to be distinct from (all markers: Supplementary Figures S1–S5) and not sister to the *Euphrasia-Rhinanthus* clade (all markers except AT1G04780: Supplementary Figure S4).

*Odontites* (including *Macrosyringion*, where available) was inferred as monophyletic by three markers (the PPR gene AT1G09680, AT1G14610, and *Atg1*) with high support (BS 97–100, PP 1; Supplementary Figures S1, S3, S5), but not by the other two markers. Here, *Odontites* was either inferred as paraphyletic due to the, yet unsupported, inclusion of *Melampyrum* (the second PPR gene AT2G37230; Supplementary Figure S2) or as polyphyletic due to the, yet unsupported, placements of *Macrosyringion*, *Nothobartsia*, *Odontitella*, and *Parentucellia* (AT1G04780; Supplementary Figure S4). With the exception of the first PPR gene AT1G09680 (Supplementary Figure S1), relationships among *Odontites* species were poorly resolved and usually insufficiently supported (Supplementary Figures S2–S5). *Nothobartsia* and *Odontitella* were inferred as sister groups (BS 65–100, PP 0.97–1) in all but two of the shorter markers (AT1G14610 and *Atg1*; Supplementary Figures S3, S5).

### Concatenated Markers

**FIGURE 3:**
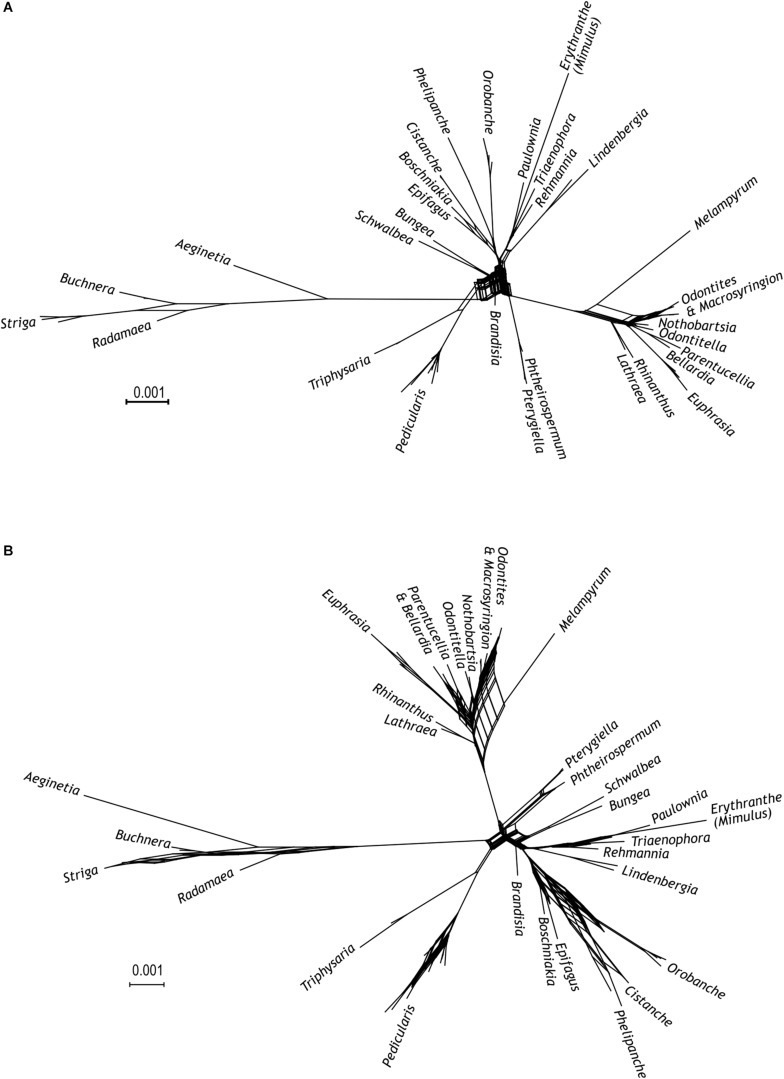
Super network from the maximum likelihood trees. Input trees are from **(A)** each of the five newly sequenced markers (AT1G09680, AT2G37230, AT1G14610, AT1G04780, and *Agt1*) and **(B)** from each of the ten used markers (five newly sequenced markers and *PHYA*, *PHYB*, ITS, *matK*, and *rps2*).

Following a supermatrix approach, we combined the five markers newly generated here. The thus combined data set comprised 4,437 nucleotide sites in 56 species. Whereas all previously identified major clades (including the *Orobanche* clade), the *Rehmannia-Triaenophora* clade, and the *Pterygiella* clade were recovered with high support (BS 98–100, PP 1), relationships among some of these clades were less certain (Figure 1). Possibly, this is due to conflicts among the genes, e.g., between the two PPR genes mentioned in the previous section, which is reflected in the network connecting major lineages of Orobanchaceae in the super network (Figure 3A). Major uncertainty was reflected by low support for a clade comprising the *Cymbaria-Siphonostegia* clade, the *Pterygiella* clade, the *Euphrasia-Rhinanthus* clade, and the *Striga-Alectra* clade (BS < 50, PP < 0.95) and for the node joining this clade with the *Castilleja-Pedicularis* clade (BP = 52, PP = 0.96; Figure 1). The *Orobanche* clade was well supported as sister to the remaining parasitic taxa (BS 100, PP 1), as were the remaining nodes uniting *Lindenbergia* and the parasitic taxa, and the node uniting these with *Rehmannia* and *Triaenophora* (BS 99–100, PP 1, Figure 1). *Nothobartsia* and *Odontitella* were inferred as sister taxa (BS 99, PP 1) well separated from *Odontites* (Figure 1). *Odontites* was inferred as monophyletic, but only from maximum likelihood and without support (BS 56), with *Macrosyringion* as sister (BS 100, PP 1).

Combining the newly developed loci with the five loci of McNeal et al. (2013) resulted in a matrix comprising 11,093 nucleotide sites from 56 species. All previously identified major clades (including the *Orobanche* clade), the *Rehmannia-Triaenophora* clade, and the *Pterygiella* clade were recovered with (nearly) maximum support (BS 99–100, PP 1; Figure 2). Relationships among major clades tended to reflect those recovered by McNeal et al. (2013), rather than relationships inferred by analyses of the five newly developed markers. Specifically, the *Striga-Alectra* clade was inferred as well-supported sister (BS 99, PP 1) to the *Castilleja-Pedicularis* clade (Figure 2) instead of to the *Euphrasia-Rhinanthus* clade (BS 81, PP 1; Figure 1) and the *Pterygiella* clade was inferred as sister to the *Euphrasia-Rhinanthus* clade (BS 98, PP 1; Figure 2) instead of to the clade comprising the *Euphrasia-Rhinanthus* clade and the *Striga-Alectra* clade (BS 70, PP 1; Figure 1). *Brandisia* was placed as sister to the clade comprising the *Striga-Alectra* clade, the *Castilleja-Pedicularis* clade, the *Euphrasia-Rhinanthus* clade, and the *Pterygiella* clade (BS 83, PP 1; Figure 2), whereas from the five marker analyses the relationship of *Brandisia* to any of these clades remained unclear (Figure 1). The *Cymbaria-Siphonostegia* clade was inferred as sister to all other hemiparasitic Orobanchaceae, yet this did not receive strong support (BS 69, PP 1; Figure 2). With respect to the phylogenetic positions of the *Orobanche* clade, the *Lindenbergia* clade, and the *Rehmannia-Triaenophora* clade as subsequent sisters to the hemiparasitic clades, the ten marker analyses agreed with the five marker analyses, yet with higher support (BS 94–100, PP 1; Figure 2). Despite the often-high bootstrap support values, there was considerable incongruence among markers with respect to phylogenetic relationships, as is reflected in reticulate relationships among major lineages in the super network including all ten markers (Figure 3B).

Ancestral character state reconstruction suggested that parasitism (i.e., hemiparasitism) evolved only once in the sister of the *Lindenbergia* clade (Figure 2). The ancestor of the clade including all parasitic taxa was inferred to be hemiparasitic (Figure 2).

## Discussion

### Phylogenetic Utility of PPR Genes and Three LCN Loci in Orobanchaceae

Analyses of two PPR genes, AT1G09680 and AT2G37230, indicated resolved, though not necessarily well-supported, relationships among major clades of Orobanchaceae and among *Odontites* species (Supplementary Figures S1, S2). This confirms the high potential of PPR genes for molecular phylogenetic studies from the family to the species level (Yuan et al., 2010; Barkan and Small, 2014; Crowl et al., 2014), notwithstanding issues of incongruence among markers from the level of major clades to the infrageneric level, as in *Odontites* (Supplementary Figures S1, S2). In line with decreasing length and concomitantly decreasing number of informative sites (measured here as number of parsimony-informative sites: Table 3), phylogenetic resolution and support were lower, especially at the backbone, in inferences from the LCN loci AT1G14610 and AT1G04780 (Supplementary Figures S3, S4), coding for an aminoacyl-tRNA ligase and an ankyrin repeat family protein, respectively (Isner et al., 2012; Ometto et al., 2012), and especially from the LCN locus *Agt1* (Supplementary Figure S5), encoding a peroxisomal photorespiratory enzyme (Liepman and Olsen, 2003). Whereas the readily amplifiable and alignable AT1G14610 and AT1G04780, to our knowledge, have not previously been used for phylogenetic purposes, *Agt1* has been suggested as phylogenetically useful locus (Li M. et al., 2008; López-Pujol et al., 2012; Gonzalez, 2014), an assessment that is not supported by our analyses of Orobanchaceae.

Practical limitations of LCN loci as used here include the difficulty in designing primers and in obtaining reliable amplification. Here, screening of more than 200 loci resulted in identification of only a few that could be used over the desired broad taxonomic range. Even some PPR genes successfully used by Yuan et al. (2010) failed to work in Orobanchaceae. Reasons for this are unclear, but may include poor primer match due to the phylogenetic distance between Verbenaceae and Orobanchaceae, evolutionary rate variation, or pseudogene formation in Orobanchaceae. It can, however, be expected that enrichment procedures, such as target-capture (Lemmon and Lemmon, 2013; Zimmer and Wen, 2015; Johnson et al., 2019) will essentially eliminate the need for the time-consuming search for suitable loci (a pipeline for identifying loci amenable to target-capture in Orobanchaceae has been suggested recently: Li et al., 2017). Using phylogenomic approaches with hundreds of loci is also expected to help resolve phylogenetic relationships in the presence of incongruence among loci (Buddenhagen et al., 2016; Crowl et al., 2017; Léveillé-Bourret et al., 2018), as long as heterogeneous population-genetic processes are taken into account (Bravo et al., 2019).

### Phylogenetic Relationships Among Major Clades

Although there are conflicts among phylogenies generated using different markers and their combinations (Figures 1–3 and Supplementary Figures S1–S5; McNeal et al., 2013), circumscription of major clades as identified previously (Bennett and Mathews, 2006; McNeal et al., 2013) is mostly confirmed from single marker analyses (Supplementary Figures S1–S5) and is well-supported from the combined data (Figures 1, 2). This is also the case for *Brandisia*, which is corroborated as a distinct lineage. The only modification to the circumscription of major clades concerns the *Pterygiella* clade [represented by *P. tenuisectum* (*Pterygiella tenuisecta*), *Pterygiella cylindrica*, and *Pt. duclouxii*], comprising *Pterygiella*, *Phtheirospermum* (except *Ph. japonicum*), and *Xizangia* (Yu et al., 2018). This small group was inferred as sister to the *Euphrasia-Rhinanthus* clade by McNeal et al. (2013), a relationship also supported by the combined 10-marker data set (Figure 2). This position is, however, found neither by the newly sequenced markers (except AT1G04780; Supplementary Figure S4), analyzed individually (Supplementary Figures S1–S3, S5) or jointly (Figure 1), nor by ITS and plastid data used by Yu et al. (2018). A closer relationship of the *Pterygiella* clade to *Lindenbergia* and *Brandisia*, as suggested by fruit and seed characters (Dong et al., 2015), is not supported by the nuclear data, but, with respect to *Brandisia*, by plastid data (Yu et al., 2018). Given these uncertainties and the deep divergence of *Pterygiella* and relatives from the *Euphrasia-Rhinanthus* clade, even if inferred as sister taxa (Figure 2), we consider it prudent to recognize this small group of East Asian genera as the *Pterygiella* clade distinct from the *Euphrasia-Rhinanthus* clade until its precise position within the family has been ascertained.

In contrast to the generally well-supported circumscription of major clades, phylogenetic relationships among these clades are not consolidated yet (Figure 3). One such area of uncertainty concerns relationships among the *Castilleja-Pedicularis* clade, the *Euphrasia-Rhinanthus* clade, and the *Striga-Alectra* clade. Bennett and Mathews (2006), using *PHYA* including obvious paralogs, inferred the *Striga-Alectra* clade as sister to the *Euphrasia-Rhinanthus* clade (with bootstrap support of at least 80), together being sister-group to the *Castilleja-Pedicularis* clade (with maximum bootstrap support). In contrast, McNeal et al. (2013), using, among others, *PHYA* excluding obvious paralogs, found the *Striga-Alectra* clade to be sister to the *Castilleja-Pedicularis* clade (with bootstrap support of at least 99 in analyses of *PHYA* and *PHYB* separately as well as combined in their 5-marker dataset), jointly being sister to the *Euphrasia-Rhinanthus* clade (including the *Pterygiella* clade). While PPR genes (Supplementary Figures S1, S2) and our 5-marker combined dataset (Figure 1) support the hypothesis of Bennett and Mathews (2006), i.e., the sister-group relationship of the *Striga-Alectra* clade and the *Euphrasia-Rhinanthus* clade, the 10-marker combined data set (Figure 2) agrees with the hypothesis of McNeal et al. (2013), i.e., a sister-group relationship of the *Striga-Alectra* clade and the *Castilleja-Pedicularis* clade. The reasons for these conflicts are unknown, but potentially include sampling of paralogs as is evident from the large effect their inclusion has on the inferred relationships (compare the *PHYA* trees inferred by Bennett and Mathews, 2006, with those inferred by McNeal et al., 2013). Paralogs have also been reported from PPR genes (AT2G37230 has experienced a recent gene duplication in *Glandularia* and *Verbena* of Verbenaceae: Yuan et al., 2010), although copies recovered in Orobanchaceae appear to be orthologs (Supplementary Figure S2). It has been shown that already tiny subsets of large phylogenomic data sets may drive contentious relationships (Shen et al., 2017), and this might also be the case here, but additional data will be needed to test this.

The position of *Brandisia* as sister to the mostly hemiparasitic clades excluding the *Cymbaria-Siphonostegia* clade is well supported by the 10-marker combined data (Figure 2). However, the uncertain position of *Brandisia* in previous studies (Bennett and Mathews, 2006; McNeal et al., 2013) and in the newly sequenced genes, whether analyzed individually or jointly (Figure 1 and Supplementary Figures S1–S5), warrants caution with respect to its phylogenetic position.

The *Cymbaria-Siphonostegia* clade, comprising five hemiparasitic genera (ca. 20 species) distributed mainly in Eurasia (Schneeweiss, 2013), has been inferred as sister-group to all other parasitic Orobanchaceae (Bennett and Mathews, 2006; McNeal et al., 2013). Whereas its precise position remains ambiguous, PPR genes (Supplementary Figures S1, S2), the 5-marker combined (Figure 1), and the 10-marker combined analyses (Figure 2) suggest that the *Cymbaria-Siphonostegia* clade is sister to, or even nested among, the mostly hemiparasitic clades (*Brandisia*, *Castilleja-Pedicularis* clade, *Euphrasia-Rhinanthus* clade, *Pterygiella* clade, *Striga-Alectra* clade). A consequence of the altered position of the *Cymbaria-Siphonostegia* clade is that the exclusively holoparasitic and, except for the shortest markers used (Supplementary Figures S4, S5), well-supported *Orobanche* clade is sister to all other parasitic clades (Figures 1, 2). Although this may suggest that holoparasitism evolved early in parasitic Orobanchaceae, conservation of the chlorophyll synthesis in holoparasitic *Phelipanche* (Wickett et al., 2011) despite loss of photosynthesis and the concomitant reductions in the plastid genome (Wicke et al., 2013, 2016) may be interpreted as evidence for a comparatively recent loss of photosynthetic functionality, i.e., a transition to holoparasitism, only in the stem lineage of the *Orobanche* clade, in line with results from ancestral character state reconstruction (Figure 2).

*Lindenbergia* is sister to the parasitic Orobanchaceae, although high support for this position is only achieved from the concatenated data sets (Figures 1, 2). The close relationship of *Lindenbergia* to parasitic lineages is supported not only by molecular-phylogenetic evidence (Young et al., 1999; Olmstead et al., 2001; McNeal et al., 2013), but also by palynological and leaf stomatal closure data (Hjertson, 1995; Bennett and Mathews, 2006). Sister to *Lindenbergia* and other Orobanchaceae is the *Rehmannia-Triaenophora* clade (here represented by the newly sampled *Triaenophora shennongjiaensis* and *Rehmannia piasezkii*, Table 1) endemic to China (Chin, 1979; Li et al., 2005; Li X.D. et al., 2008). A close relationship of *Rehmannia* and/or *Triaenophora* to Orobanchaceae has been suggested before (Albach et al., 2009; Xia et al., 2009), which eventually has led to the extension of Orobanchaceae to include both genera (Angiosperm Phylogeny Group, 2016).

## Conclusion

We analyzed the potential of five nuclear genes (two PPR genes and three LCN genes) to address phylogenetic relationships within Orobanchaceae focusing on major clades identified previously. Of those, the longer markers (the two PPR genes, AT1G09680 and AT2G37230, and the LCN locus AT1G04780) consistently performed better in inferring relationships within and among major clades than the two short markers (LCN loci AT1G14610 and *Agt1*). Whereas extension of the data set (increasing sequence length by adding more loci) clearly improves resolving power, at least when concatenating loci, and corroborates and refines circumscription of major clades, this study also highlights the limits of sequencing hand-picked loci for phylogenetic purposes. These are, among others, the large effort to establish suitable nuclear loci and the inability to deal with incongruence among loci through species tree estimation methods as these methods cannot be applied because of the too low number of sequenced loci. We expect that already available phylogenomic approaches, once applied to Orobanchaceae, will help to resolve relationships among major clades. This notwithstanding, congruence among markers in inference of major clades of Orobanchaceae allows these major clades to be taken as frameworks for detailed, species-level, phylogenetic studies in this family, a model for studying plant parasitism.

## Data Availability

Newly generated sequences are available in GenBank under accession numbers MK588398–MK588632. Data matrices are available at Dryad under doi: 10.5061/dryad.31cf160.

## Author Contributions

GS and XL designed the study. XL and TF generated and analyzed the data. XL and GS drafted the manuscript. XL, CR, and GS finalized the manuscript. All authors read and approved the final version of the manuscript.

## Conflict of Interest Statement

The authors declare that the research was conducted in the absence of any commercial or financial relationships that could be construed as a potential conflict of interest.

## Footnotes

1 http://ppgp.huck.psu.edu/blast.php

2 http://ppgp.huck.psu.edu/

3 https://sites.google.com/a/ualberta.ca/onekp/

4 https://phytozome.jgi.doe.gov/pz/portal.html#

## Supplementary Material

FIGURE S1 Phylogenetic relationships within Orobanchaceae inferred using maximum likelihood on an AT1G09680 data set. Numbers at branches are maximum likelihood bootstrap support values of at least 50 and, in italics, posterior probabilities of at least 0.95. Circumscription of major clades within Orobanchaceae is indicated.

FIGURE S2 Phylogenetic relationships within Orobanchaceae inferred using maximum likelihood on an AT2G37230 data set. Numbers at branches are maximum likelihood bootstrap support values of at least 50 and, in italics, posterior probabilities of at least 0.95. Circumscription of major clades within Orobanchaceae is indicated.

FIGURE S3 Phylogenetic relationships within Orobanchaceae inferred using maximum likelihood on an AT1G14610 data set. Numbers at branches are maximum likelihood bootstrap support values of at least 50 and, in italics, posterior probabilities of at least 0.95. Circumscription of major clades within Orobanchaceae is indicated.

FIGURE S4 Phylogenetic relationships within Orobanchaceae inferred using maximum likelihood on an AT1G04780 data set. Numbers at branches are maximum likelihood bootstrap support values of at least 50 and, in italics, posterior probabilities of at least 0.95. Circumscription of major clades within Orobanchaceae is indicated.

FIGURE S5 Phylogenetic relationships within Orobanchaceae inferred using maximum likelihood on an *Agt1* data set. Numbers at branches are maximum likelihood bootstrap support values of at least 50 and, in italics, posterior probabilities of at least 0.95. Circumscription of major clades within Orobanchaceae is indicated.

TABLE S1 List of taxa, locality and voucher information, and sequence source (GenBank accession numbers or other databases).

TABLE S2 List of low-copy nuclear genes successfully PCR-amplified in up to 27 taxa.

## References

[B1] Akaike H. (1974). A new look at the statistical model identification. *IEEE Trans. Automatic Control* 19 716–723. 10.1109/TAC.1974.1100705

[B2] Albach D. C. Yan K. Jensen S. R. Li H.-Q. (2009). Phylogenetic placement of *Triaenophora* (formerly *Scrophulariaceae*) with some implications for the phylogeny of Lamiales. *Taxon* 58 749–756. 10.1002/tax.583005

[B3] Álvarez I. Wendel J. F. (2003). Ribosomal ITS sequences and plant phylogenetic inference. *Mol. Phylogen. Evol.* 29 417–434. 10.1016/S1055-7903(03)00208-2 14615184

[B4] Angiosperm Phylogeny Group (2016). An update of the angiosperm phylogeny group classification for the orders and families of flowering plants: APG IV. *Bot. J. Linn. Soc.* 181 1–20. 10.1111/boj.12385 26027755

[B5] Babineau M. Gagnon E. Bruneau A. (2013). Phylogenetic utility of 19 low copy nuclear genes in closely related genera and species of caesalpinioid legumes. *South Afr. J. Bot.* 89 94–105. 10.1016/j.sajb.2013.06.018

[B6] Barkan A. Small I. (2014). Pentatricopeptide repeat proteins in plants. *Annu. Rev. Plant Biol.* 65 415–442. 10.1146/annurev-arplant-050213-040159 24471833

[B7] Beck-Mannagetta G. (1930). “Orobanchaceae,” in *Das Pflanzenreich IV 261*, ed. Engler A. (Leipzig: Wilhelm Engelmann), 1–348.

[B8] Bennett J. Mathews S. (2006). Phylogeny of the parasitic plant family Orobanchaceae inferred from phytochrome A. *Am. J. Bot.* 93 1039–1051. 10.3732/ajb.93.7.1039 21642169

[B9] Bravo G. A. Antonelli A. Bacon C. D. Bartoszek K. Blom M. P. K. Huynh S. (2019). Embracing heterogeneity: coalescing the Tree of Life and the future of phylogenomics. *PeerJ* 7:e6399. 10.7717/peerj.6399 30783571 PMC6378093

[B10] Buddenhagen C. Lemmon A. R. Lemmon E. M. Bruhl J. Cappa J. Clement W. L. (2016). Anchored phylogenomics of angiosperms I: assessing the robustness of phylogenetic estimates. *bioRxiv* [Preprint]. 10.1101/086298

[B11] Chin T. L. (1979). “*Rehmannia* and *Triaenophora*,” in *Flora Reipublicae Popularis Sinicae 67 Part 2 Scrophulariaceae*, eds Tsoong P. C. Yang H. P. (Bejing: Science Press), 212–222.

[B12] Choi H. K. Luckow M. Doyle J. Cook D. R. (2006). Development of nuclear gene-derived molecular markers linked to legume genetic maps. *Mol. Genet. Genom.* 276 56–70. 10.1007/s00438-006-0118-8 16642337

[B13] Crowl A. A. Mavrodiev E. Mansion G. Haberle R. Pistarino A. Kamari G. (2014). Phylogeny of Campanuloideae (Campanulaceae) with emphasis on the utility of nuclear pentatricopeptide repeat (PPR) genes. *PLoS One* 9:e94199. 10.1371/journal.pone.0094199 24718519 PMC3981779

[B14] Crowl A. A. Myers C. Cellinese N. (2017). Embracing discordance: phylogenomic analyses provide evidence for allopolyploidy leading to cryptic diversity in a Mediterranean *Campanula* (Campanulaceae) clade. *Evolution* 71 913–922. 10.1111/evo.13203 28186341 PMC5413844

[B15] Cubero J. I. Moreno M. T. (1996). “Parasitic plant science: a quarter century,” in *Advances in Parasitic Plant Research*, eds Moreno M. T. Cubero J. I. Berner D. Joel D. Musselman L. J. Parker C. (Sevilla: Consejería de Agricultura y Pesca, Junta de Andalucía), 15–21.

[B16] dePamphilis C. W. Palmer J. D. (1990). Loss of photosynthetic and chlororespiratory genes from the plastid genome of a parasitic flowering plant. *Nature* 348 337–339. 10.1038/348337a0 2250706

[B17] dePamphilis C. W. Young N. D. Wolfe A. D. (1997). Evolution of plastid gene *rps2* in a lineage of hemiparasitic and holoparasitic plants: many losses of photosynthesis and complex patterns of rate variation. *Proc. Natl. Acad Sci. U.S.A.* 94 7367–7372. 10.1073/pnas.94.14.7367 9207097 PMC23827

[B18] Dong L.-N. Wang H. Wortley A. H. Li D. Z. Lu L. (2015). Fruit and seed morphology in some representative genera of tribe Rhinantheae sensu lato (Orobanchaceae) and related taxa. *Plant Syst. Evol.* 301 479–500. 10.1007/s00606-014-1087-8

[B19] Duarte J. M. Wall P. K. Edger P. P. Landherr L. L. Ma H. Pires J. C. (2010). Identification of shared single copy nuclear genes in *Arabidopsis*, *Populus*, *Vitis* and *Oryza* and their phylogenetic utility across various taxonomical levels. *BMC Evol. Biol.* 10:61–79. 10.1186/1471-2148-10-61 20181251 PMC2848037

[B20] Edgar R. C. (2004). MUSCLE: multiple sequence alignment with high accuracy and high throughput. *Nucl. Acids Res.* 32 1792–1797. 10.1093/nar/gkh340 15034147 PMC390337

[B21] Fischer E. (2004). “Scrophulariaceae,” in *The Families and Genera of Vascular Plants VII: Flowering Plants, Dicotyledons, Lamiales (Excluding Acanthaceae, Including Avicenniaceae)*, ed. Kadereit J. W. (Berlin: Springer), 333–432.

[B22] Gaudeul M. Siljak-Yakovlev S. Jang T. S. Rouhan G. (2018). Reconstructing species relationships within the recently diversified genus *Odontites* Ludw. (*Orobanchaceae*): evidence for extensive reticulate evolution. *Int. J. Plant Sci.* 179 1–20. 10.1086/694763

[B23] Gonzalez L. A. (2014). *Phylogenetics and Mating System Evolution in the Southern South American Valeriana (Valerianaceae).* Master’s thesis,University of New Orleans: New Orleans, LO.

[B24] Hall T. A. (1999). BioEdit: a user-friendly biological sequence alignment editor and analysis program for Windows 95/98/NT. *Nucl. Acids Symp. Ser.* 41 95–98.

[B25] Hellsten U. Wright K. M. Jenkins J. Shu S. Yuan Y. Wessler S. R. (2013). Fine-scale variation in meiotic recombination in *Mimulus* inferred from population shotgun sequencing. *Proc. Natl. Acad. Sci. U.S.A.* 110 19478–19482. 10.1073/pnas.1319032110 24225854 PMC3845195

[B26] Hjertson M. L. (1995). Taxonomy, phylogeny, and biogeography of *Lindenbergia* (Scrophulariaceae). *Bot. J. Linn. Soc.* 119 265–321. 10.1016/S0024-4074(95)80002-6

[B27] Huson D. H. Bryant D. (2006). Application of phylogenetic networks in evolutionary studies. *Mol. Biol. Evol.* 23 254–267. 10.1093/molbev/msj030 16221896

[B28] Huson D. H. Dezulian T. Kloepper T. Steel M. A. (2004). Phylogenetic super-networks from partial trees. *IEEE ACM Trans. Comp. Biol. Bioinform.* 1 151–158. 10.1109/TCBB.2004.44 17051697

[B29] Isner J. C. Nuhse T. Maathuis F. J. (2012). The cyclic nucleotide cGMP is involved in plant hormone signalling and alters phosphorylation of *Arabidopsis thaliana* root proteins. *J. Exp. Bot.* 63 3199–3205. 10.1093/jxb/ers045 22345640 PMC3350932

[B30] Joel D. M. Gressel J. Musselman L. J. (eds) (2013). *Parasitic Orobanchaceae. Parasitic Mechanisms and Control Strategies.* Berlin: Springer.

[B31] Johnson M. Pokorny L. Dodsworth S. Botigue L. R. Cowan R. S. Devault A. (2019). A universal probe set for targeted sequencing of 353 nuclear genes from any flowering plant designed using k-medoids clustering. *Syst. Biol.* 68 594–606. 10.1093/sysbio/syy086 30535394 PMC6568016

[B32] Kuijt J. (1969). *The Biology of Parasitic Flowering Plants.* Berkeley, CA: University of California Press.

[B33] Lanfear R. Calcott B. Ho S. Y. Guindon S. (2012). PartitionFinder: combined selection of partitioning schemes and substitution models for phylogenetic analyses. *Mol. Biol. Evol.* 29 1695–1701. 10.1093/molbev/mss020 22319168

[B34] Latvis M. Jacobs S. J. Mortimer S. M. E. Richards M. Blischak P. D. Mathews S. (2017). Primers for *Castilleja* and their utility across Orobanchaceae: II. single-copy nuclear loci. *Appl. Plant Sci.* 5:1700038. 10.3732/apps.1700038 28989822 PMC5628026

[B35] Lemmon E. M. Lemmon A. R. (2013). High-throughput genomic data in systematics and phylogenetics. *Annu. Rev. Ecol. Evol. Syst.* 44 99–121. 10.1146/annurev-ecolsys-110512-135822

[B36] Léveillé-Bourret É. Starr J. R. Ford B. A. Lemmon E. M. Lemmon A. R. (2018). Resolving rapid radiations within angiosperm families using anchored phylogenomics. *Syst. Biol.* 67 94–112. 10.1093/sysbio/syx050 28472459

[B37] Li M. Wunder J. Bissoli G. Scarponi E. Gazzani S. Barbaro E. (2008). Development of COS genes as universally amplifiable markers for phylogenetic reconstructions of closely related plant species. *Cladistics* 24 727–745. 10.1111/j.1096-0031.2008.00207.x

[B38] Li X. Hao B. Pan D. Schneeweiss G. M. (2017). Marker development for phylogenomics: the case of *Orobanchaceae*, a plant family with contrasting nutritional modes. *Front. Plant Sci.* 8:1973. 10.3389/fpls.2017.01973 29218053 PMC5704539

[B39] Li X. Jang T.-S. Temsch E. M. Kato H. Takayama K. Schneeweiss G. M. (2016). Molecular and karyological data confirm that the enigmatic genus *Platypholis* from Bonin-Islands (SE Japan) is phylogenetically nested within *Orobanche* (Orobanchaceae). *J. Plant Res.* 130 273–280. 10.1007/s10265-016-0888-y 28004281 PMC5318490

[B40] Li X. D. Li J. Q. Zan Y. Y. (2005). A new species of *Triaenophora* (Scrophulariaceae) from China. *Novon* 15 559–561.

[B41] Li X. D. Zan Y. Y. Li J. Q. Yang S. Z. (2008). A numerical taxonomy of the genera *Rehmannia* and *Triaenophora* (Scrophulariaceae). *J. Syst. Evol.* 46 730–737.

[B42] Liepman A. H. Olsen L. J. (2003). Alanine aminotransferase homologs catalyze the glutamate:glyoxylate aminotransferase reaction in peroxisomes of *Arabidopsis*. *Plant Physiol.* 131 215–227. 10.1104/pp.011460 12529529 PMC166801

[B43] López-Pujol J. Garcia-Jacas N. Susanna A. Vilatersana R. (2012). Should we conserve pure species or hybrid species? Delimiting hybridization and introgression in the Iberian endemic *Centaurea podospermifolia*. *Biol. Conserv.* 152 271–279. 10.1016/j.biocon.2012.03.032

[B44] Maddison W. P. Maddison D. R. (2018). *Mesquite: a Modular System for Evolutionary Analysis. Version 3.51.* Available at: http://www.mesquiteproject.org (accessed April 29, 2019).

[B45] Matasci N. Hung L. H. Yan Z. Carpenter E. J. Wickett N. J. Mirarab S. (2014). Data access for the 1,000 Plants (1KP) project. *GigaScience* 3:17. 10.1186/2047-217X-3-17 25625010 PMC4306014

[B46] McNeal J. Bennett J. R. Wolfe A. D. Mathews S. (2013). Phylogeny and origins of holoparasitism in Orobanchaceae. *Am. J. Bot.* 100 971–983. 10.3732/ajb.1200448 23608647

[B47] McWilliam H. Li W. Uludag M. Squizzato S. Park Y. M. Buso N. (2013). Analysis tool web services from the EMBL-EBI. *Nucl. Acids Res.* 41 W597–W600. 10.1093/nar/gkt376 23671338 PMC3692137

[B48] Nickrent D. L. (2019). *The Parasitic Plant Connection.* Available at: https://parasiticplants.siu.edu/ (accessed April 12, 2019).

[B49] Nickrent D. L. Duff R. J. Colwell A. E. Wolfe A. D. Young N. D. Steiner K. E. (1998). “Molecular phylogenetic and evolutionary studies of parasitic plants,” in *Molecular Systematics of Plants II*, eds Soltis D. E. Soltis P. S. Doyle J. J. (Boston, MA: Springer), 211–241. 10.1007/978-1-4615-5419-6_8

[B50] Olmstead R. G. dePamphilis C. W. Wolfe A. D. Young N. D. Elison W. J. Reeves P. A. (2001). Disintegration of the Scrophulariaceae. *Am. J. Bot.* 88 348–362. 10.2307/2657024 11222255

[B51] Ometto L. Li M. Bresadola L. Varotto C. (2012). Rates of evolution in stress-related genes are associated to habitat preference in two *Cardamine* lineages. *BMC Evol. Biol.* 12:7. 10.1186/1471-2148-12-7 22257588 PMC3398273

[B52] O’Toole N. Hattori M. Andres C. Iida K. Lurin C. Schmitz-Linneweber C. (2008). On the expansion of the pentatricopeptide repeat gene family in plants. *Mol. Biol. Evol.* 25 1120–1128. 10.1093/molbev/msn057 18343892

[B53] Park J. M. Manen J. F. Colwell A. E. Schneeweiss G. M. (2008). A plastid gene phylogeny of the non-photosynthetic parasitic *Orobanche* (Orobanchaceae) and related genera. *J. Plant Res.* 121 365–376. 10.1007/s10265-008-0169-5 18483784

[B54] Parker C. Riches C. R. (1993). *Parasitic Weeds of the World: Biology and Control.* Wallingford: CAB International.

[B55] Pinto-Carrasco D. Scheunert A. Heubl G. Rico E. Martinez-Ortega M. M. (2017). Unravelling the phylogeny of the root-hemiparasitic genus *Odontites* (tribe Rhinantheae, Orobanchaceae): evidence for five main lineages. *Taxon* 66 886–908. 10.12705/664.6

[B56] Rambaut A. Drummond A. J. Xie D. Baele G. Suchard M. A. (2018). Posterior summarisation in Bayesian phylogenetics using Tracer 1.7. *Syst. Biol.* 67 901–904. 10.1093/sysbio/syy032 29718447 PMC6101584

[B57] Ronquist F. Huelsenbeck J. P. (2003). MrBayes 3: Bayesian phylogenetic inference under mixed models. *Bioinformatics* 19 1572–1574. 10.1093/bioinformatics/btg180 12912839

[B58] Sang T. (2002). Utility of low-copy nuclear gene sequence in plant phylogenetics. *Crit. Rev. Biochem. Mol. Biol.* 37 121–147. 10.1080/10409230290771474 12139440

[B59] Schneeweiss G. M. (2013). “Phylogenetic relationships and evolutionary trends in Orobanchaceae,” in *Parasitic Orobanchaceae: Parasitic Mechanisms and Control Strategies*, eds Joel D. M. Gressel J. Musselman L. J. (Berlin: Springer), 243–265. 10.1007/978-3-642-38146-1_14

[B60] Shen X. X. Hittinger C. T. Rokas A. (2017). Contentious relationships in phylogenomic studies can be driven by a handful of genes. *Nat. Ecol. Evol.* 1:0126. 10.1038/s41559-017-0126 28812701 PMC5560076

[B61] Stamatakis A. (2014). RAxML version 8: a tool for phylogenetic analysis and post-analysis of large phylogenies. *Bioinformatics* 30 1312–1313. 10.1093/bioinformatics/btu033 24451623 PMC3998144

[B62] Stamatakis A. Hoover P. Rougemont J. (2008). A rapid bootstrap algorithm for the RAxML web servers. *Syst. Biol.* 57 758–771. 10.1080/10635150802429642 18853362

[B63] Swofford D. L. (2002). *PAUP^*^ Phylogenetic Analysis Using Parsimony (^*^and Other Methods). Version 4b10.* Sunderland, MA: Sinauer Associates.

[B64] Wicke S. Müller K. F. dePamphilis C. W. Quandt D. Bellot S. Schneeweiss G. M. (2016). Mechanistic model of evolutionary rate variation en route to a nonphotosynthetic lifestyle in plants. *Proc. Natl. Acad. Sci. U.S.A.* 113 9045–9050. 10.1073/pnas.1607576113 27450087 PMC4987836

[B65] Wicke S. Müller K. F. dePamphilis C. W. Quandt D. Wickett N. J. Zhang Y. (2013). Mechanisms of functional and physical genome reduction in photosynthetic and nonphotosynthetic parasitic plants of the broomrape family. *Plant Cell* 25 3711–3725. 10.1105/tpc.113.113373 24143802 PMC3877813

[B66] Wickett N. J. Honaas L. A. Wafula E. K. Das M. Huang K. Wu B. (2011). Transcriptomes of the parasitic plant family Orobanchaceae reveal surprising conservation of chlorophyll synthesis. *Curr. Biol.* 21 2098–2104. 10.1016/j.cub.2011.11.011 22169535

[B67] Wolfe A. D. dePamphilis C. W. (1998). The effect of relaxed functional constraints on the photosynthetic gene rbcL in photosynthetic and nonphotosynthetic parasitic plants. *Mol. Biol. Evol.* 15 1243–1258. 10.1093/oxfordjournals.molbev.a025853 9787431

[B68] Wolfe A. D. Randle C. P. Liu L. Steiner K. E. (2005). Phylogeny and biogeography of Orobanchaceae. *Folia Geobot.* 40 115–134. 10.1007/BF02803229

[B69] Xia Z. Wang Y. Z. Smith J. F. (2009). Familial placement and relations of *Rehmannia* and *Triaenophora* (Scrophulariaceae s.l.) inferred from five gene regions. *Am. J. Bot.* 96 519–530. 10.3732/ajb.0800195 21628207

[B70] Yang Z. Wafula E. K. Honaas L. A. Zhang H. Das M. Fernandez-Aparicio M. (2015). Comparative transcriptome analyses reveal core parasitism genes and suggest gene duplication and repurposing as sources of structural novelty. *Mol. Biol. Evol.* 32 767–790. 10.1093/molbev/msu343 25534030 PMC4327159

[B71] Young N. D. Steiner K. E. dePamphilis C. W. (1999). The evolution of parasitism in Scrophulariaceae/Orobanchaceae: plastid gene sequences refute an evolutionary transition series. *Ann. Miss. Bot. Garden* 86 876–893. 10.2307/2666173

[B72] Yu W. B. Randle C. P. Lu L. Wang H. Yang J. B. dePamphilis C. W. (2018). The hemiparasitic plant *Phtheirospermum* (Orobanchaceae) is polyphyletic and contains cryptic species in the Hengduan Mountains of southwest China. *Front. Plant Sci.* 9:142. 10.3389/fpls.2018.00142 29479366 PMC5812252

[B73] Yuan Y. W. Liu C. Marx H. E. Olmstead R. G. (2009). The pentatricopeptide repeat (PPR) gene family, a tremendous resource for plant phylogenetic studies. *New Phytol.* 182 272–283. 10.1111/j.1469-8137.2008.02739.x 19192190

[B74] Yuan Y. W. Liu C. Marx H. E. Olmstead R. G. (2010). An empirical demonstration of using pentatricopeptide repeat (PPR) genes as plant phylogenetic tools: phylogeny of Verbenaceae and the *Verbena* complex. *Mol. Phylogen. Evol.* 54 23–35. 10.1016/j.ympev.2009.08.029 19733248

[B75] Zhang N. Zeng L. Shan H. Ma H. (2012). Highly conserved low-copy nuclear genes as effective markers for phylogenetic analyses in angiosperms. *New Phytol.* 195 923–937. 10.1111/j.1469-8137.2012.04212.x 22783877

[B76] Zimmer E. A. Wen J. (2012). Using nuclear gene data for plant phylogenetics: progress and prospects. *Mol. Phylogen. Evol.* 65 774–785. 10.1016/j.ympev.2012.07.015 22842093

[B77] Zimmer E. A. Wen J. (2015). Using nuclear gene data for plant phylogenetics: progress and prospects II. next-gen approaches. *J. Syst. Evol.* 53 371–379. 10.1111/jse.12174

