# Supplementary figures and images for: Phylogenetic Relationships in Orobanchaceae Inferred From Low-Copy Nuclear Genes: Consolidation of Major Clades and Identification of a Novel Position of the Non-photosynthetic Orobanche Clade Sister to All Other Parasitic Orobanchaceae
Source: Front Plant Sci. 2019 Jul 16;10:902. doi: 10.3389/fpls.2019.00902 (PMC6646720; doi:10.3389/fpls.2019.00902)

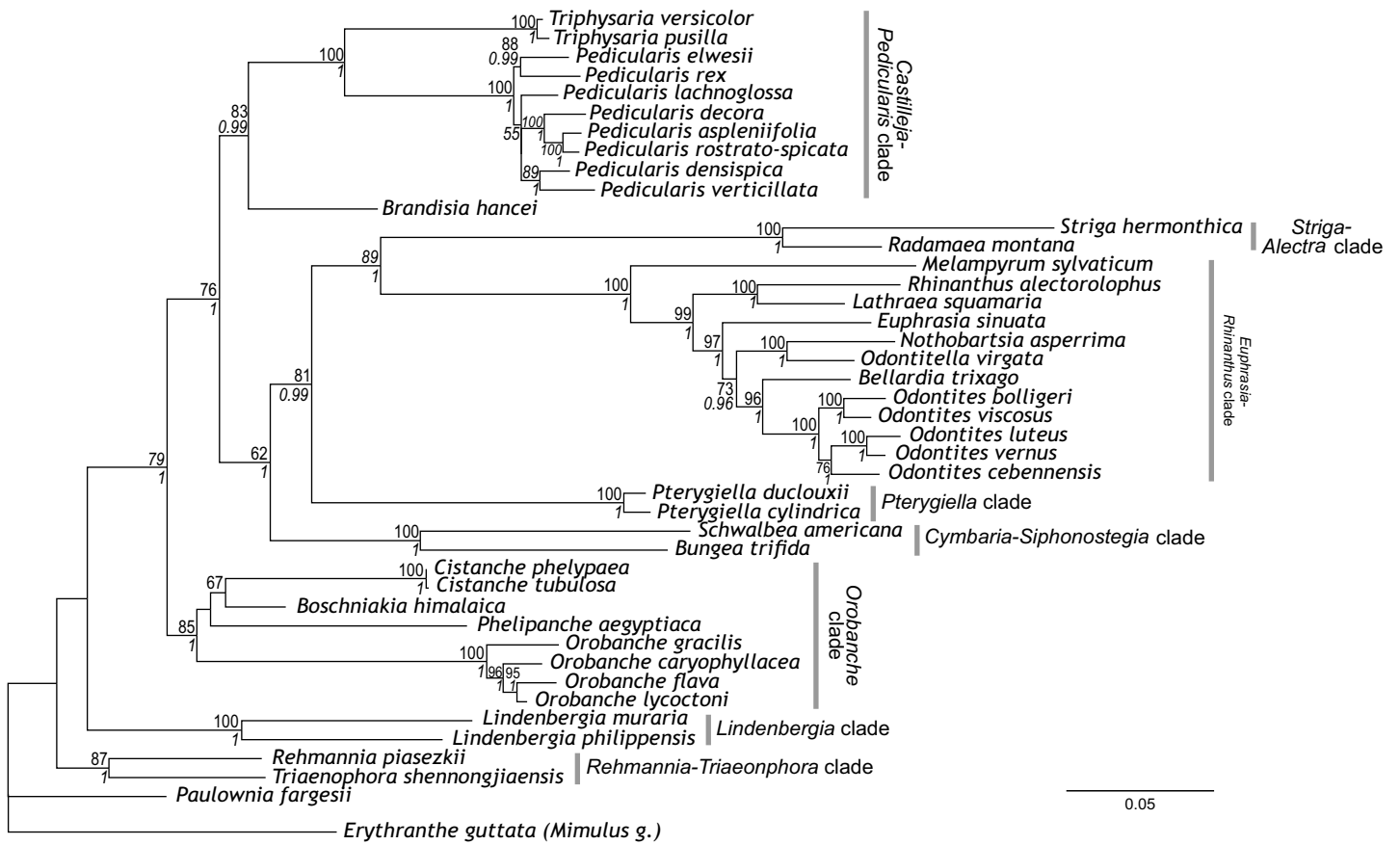

Supplement: FIGURE S1 — Phylogenetic relationships within Orobanchaceae inferred using maximum likelihood on an AT1G09680 data set. Numbers at branches are maximum likelihood bootstrap support values of at least 50 and, in italics, posterior probabilities of at least 0.95. Circumscription of major clades within Orobanchaceae is indicated. [file Data_Sheet_1.PDF]

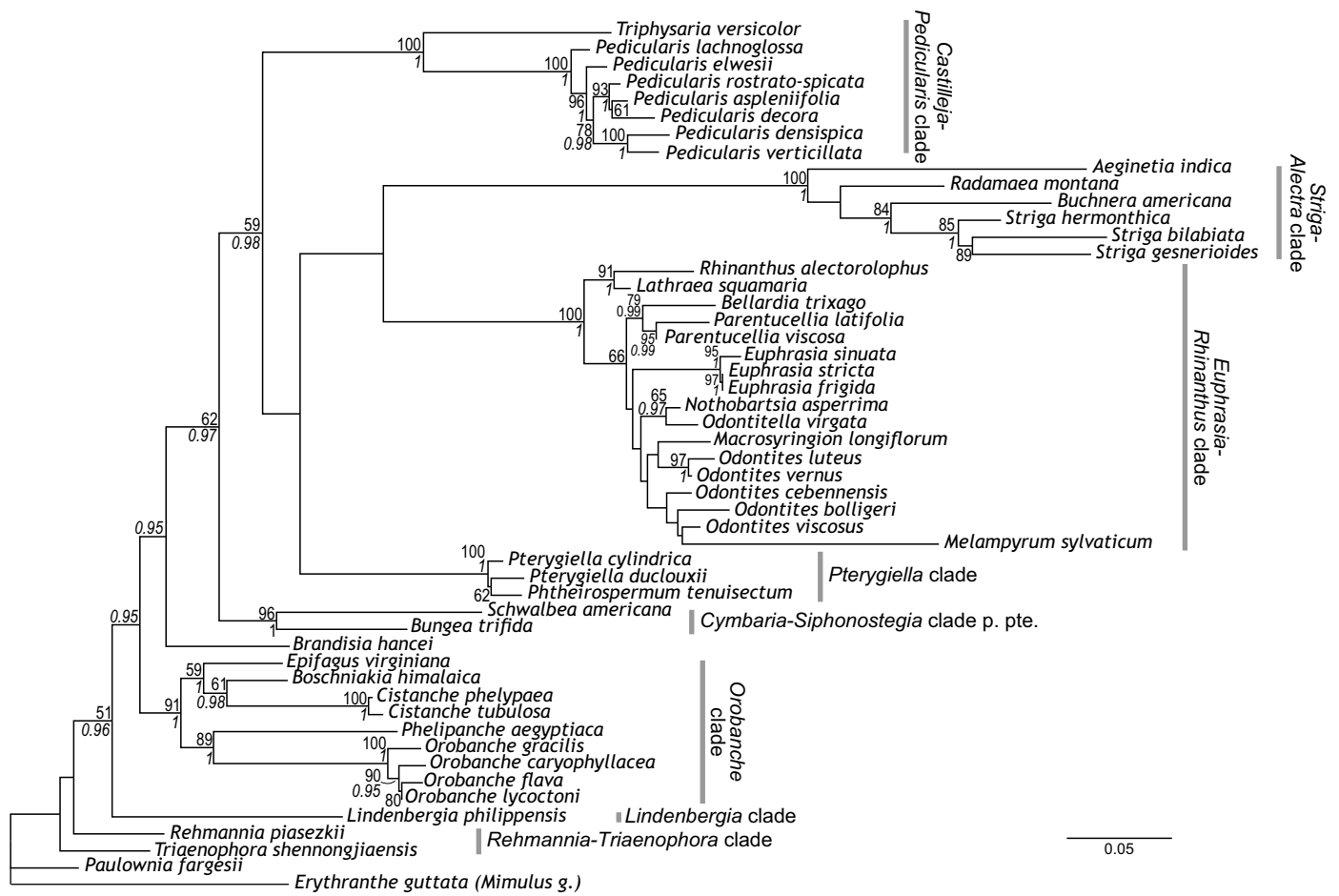

Supplement: FIGURE S2 — Phylogenetic relationships within Orobanchaceae inferred using maximum likelihood on an AT2G37230 data set. Numbers at branches are maximum likelihood bootstrap support values of at least 50 and, in italics, posterior probabilities of at least 0.95. Circumscription of major clades within Orobanchaceae is indicated. [file Data_Sheet_2.PDF]

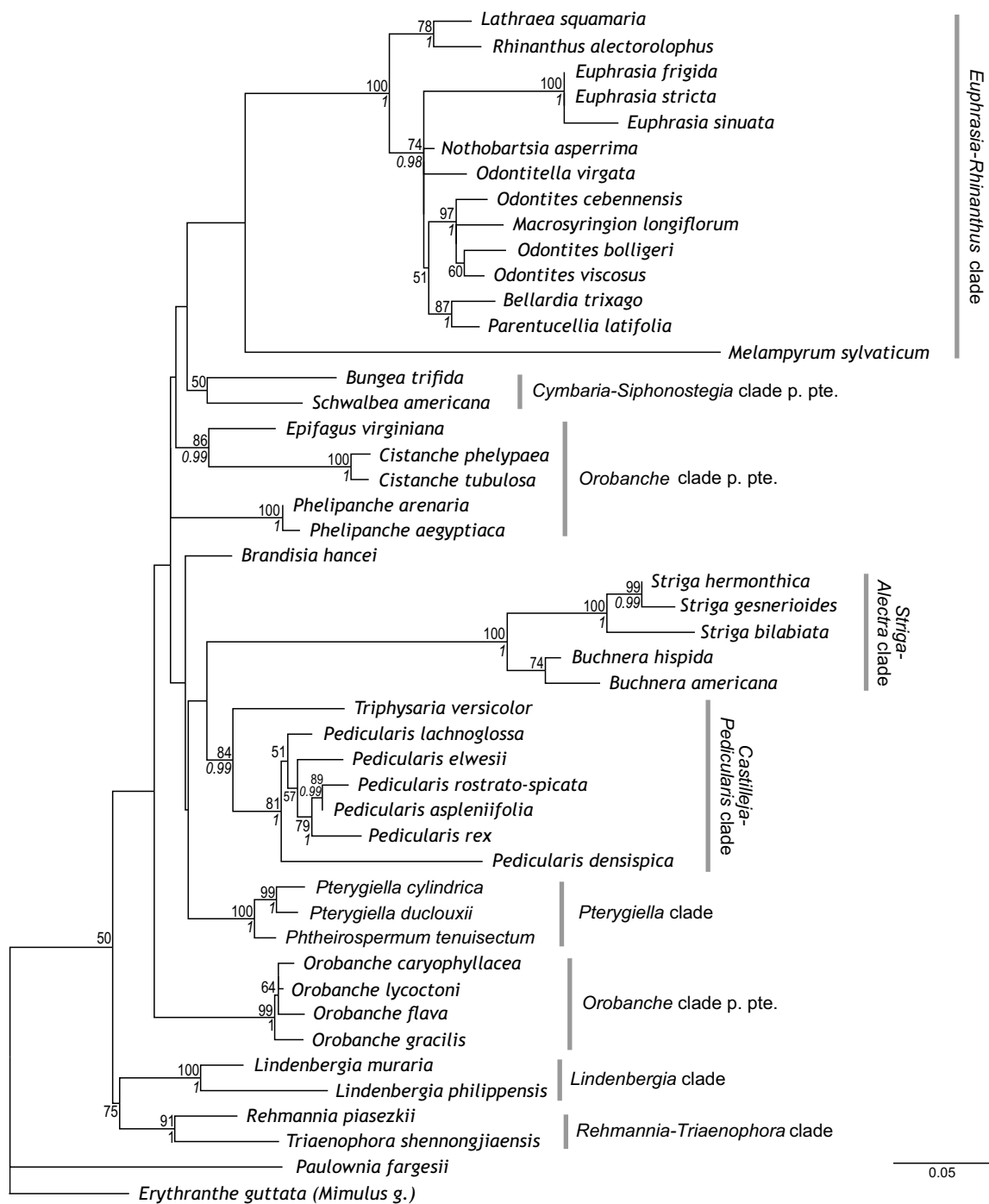

Supplement: FIGURE S3 — Phylogenetic relationships within Orobanchaceae inferred using maximum likelihood on an AT1G14610 data set. Numbers at branches are maximum likelihood bootstrap support values of at least 50 and, in italics, posterior probabilities of at least 0.95. Circumscription of major clades within Orobanchaceae is indicated. [file Data_Sheet_3.PDF]

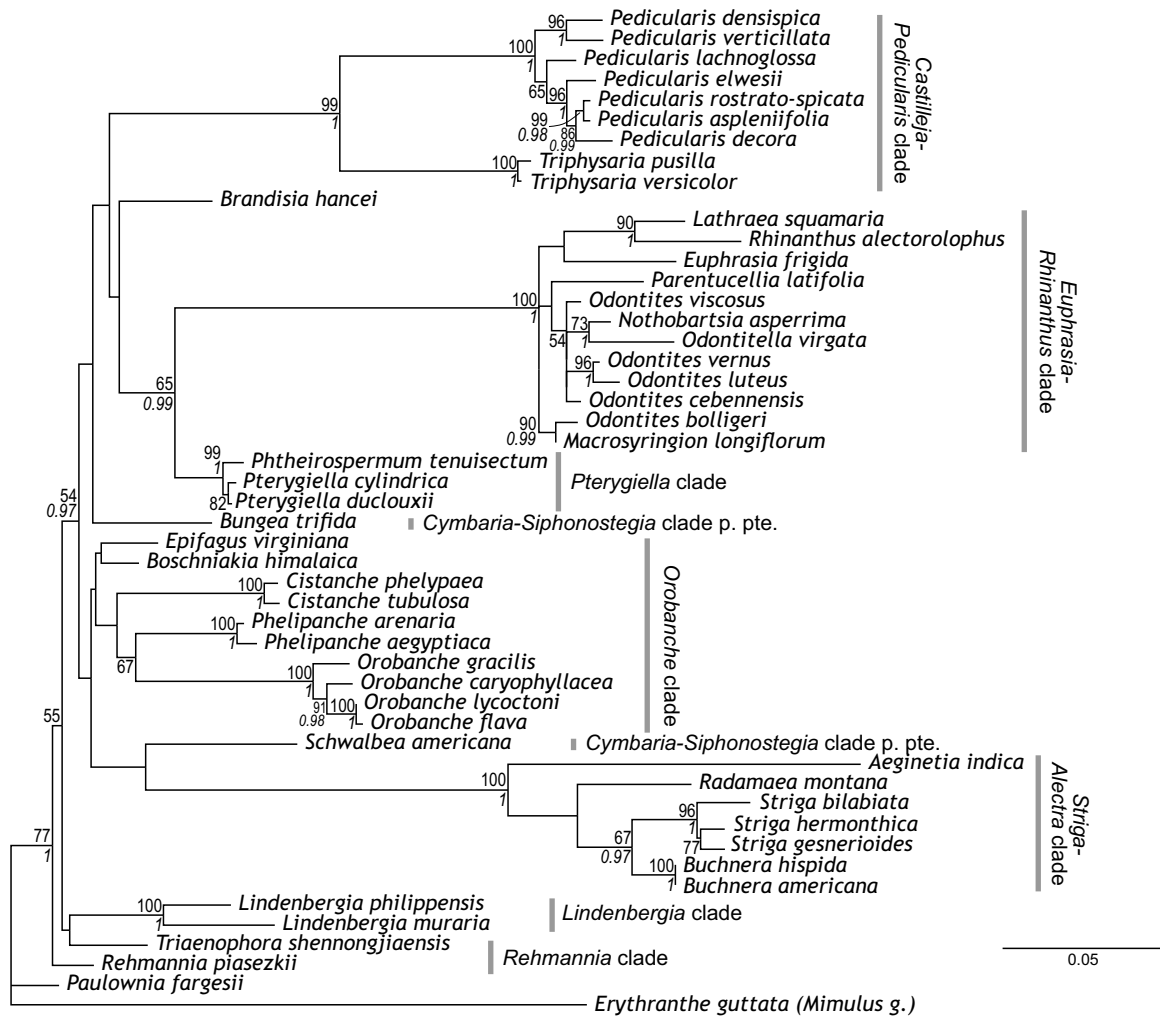

Supplement: FIGURE S4 — Phylogenetic relationships within Orobanchaceae inferred using maximum likelihood on an AT1G04780 data set. Numbers at branches are maximum likelihood bootstrap support values of at least 50 and, in italics, posterior probabilities of at least 0.95. Circumscription of major clades within Orobanchaceae is indicated. [file Data_Sheet_4.PDF]

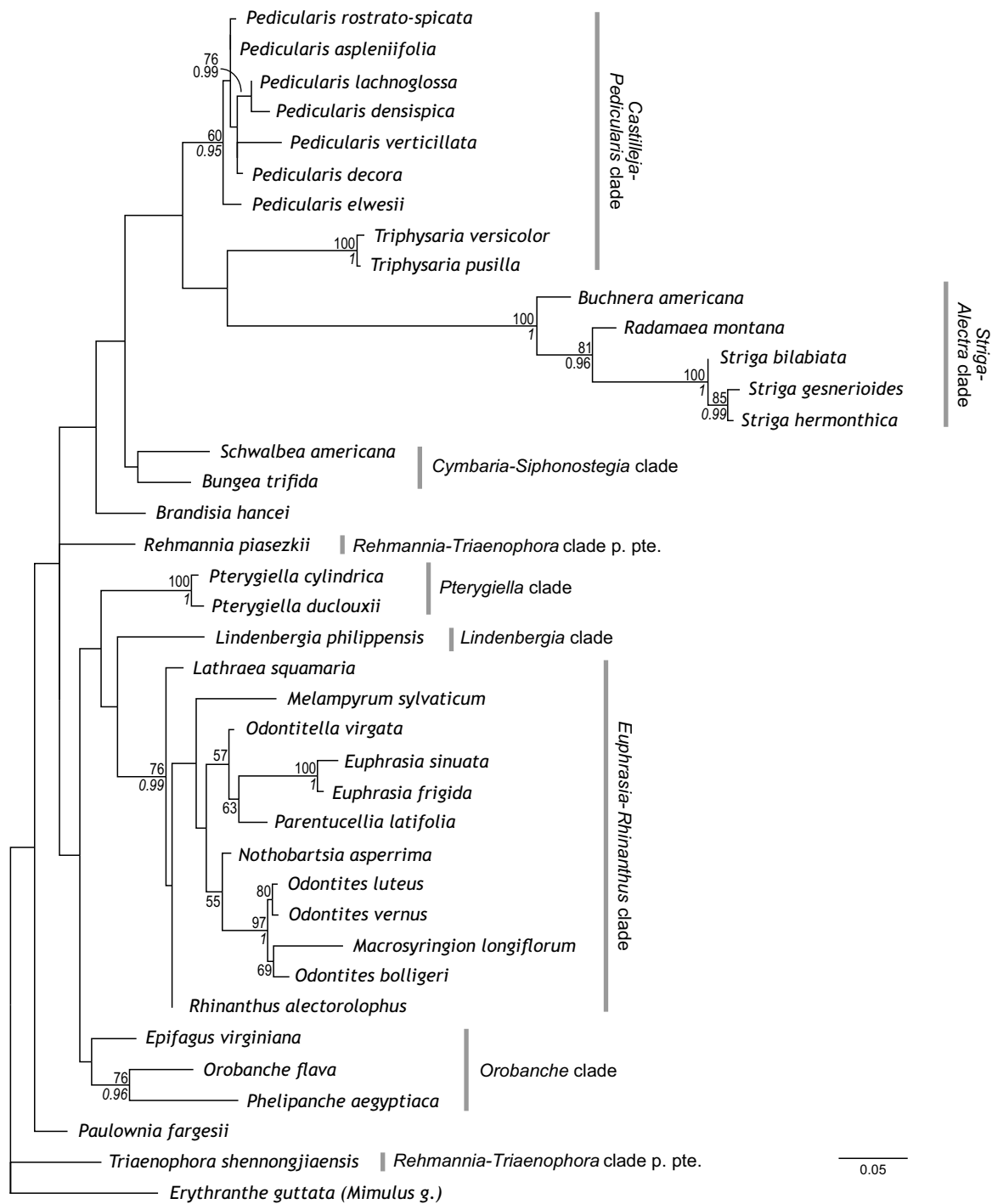

Supplement: FIGURE S5 — Phylogenetic relationships within Orobanchaceae inferred using maximum likelihood on an Agt1 data set. Numbers at branches are maximum likelihood bootstrap support values of at least 50 and, in italics, posterior probabilities of at least 0.95. Circumscription of major clades within Orobanchaceae is indicated. [file Data_Sheet_5.PDF]
